# Supplementary material for: Hippocampal volume across age: Nomograms derived from over 19,700 people in UK Biobank
Source: Neuroimage Clin. 2019 Jun 19;23:101904. doi: 10.1016/j.nicl.2019.101904 (PMC6603440; doi:10.1016/j.nicl.2019.101904)
Supplement: Supplementary file 1 — Supplementary material [file mmc1.docx]

**Supplementary material:**

Hippocampal volume across age: Nomograms derived from over 19,700 people in UK Biobank

*Lisa Nobis, Sanjay G. Manohar, Stephen M. Smith, Fidel Alfaro-Almagro, Mark Jenkinson, Clare E. Mackay, Masud Husain*

**Suppl. Table S1**: Brain volume by hypertension status and BMI correlations

**Suppl. Table S2**: Brain volume by education level

**Suppl. Table S3:** Brain volume by smoking status

**Suppl. Table S4**: Brain volume by hemisphere

**Suppl. Table S5**: Brain volume by sex

**Suppl. Table S6:** Model estimates from joinpoint regression of total grey matter

**Suppl. Figure S1**: Nomogram of left hippocampus for females, uncorrected for head size

**Suppl. Figure S2**: Nomogram of left hippocampus for males, uncorrected for head size

**Suppl. Figure S3:** Nomogram of right hippocampus for females, uncorrected for head size

**Suppl. Figure S4**: Nomogram of right hippocampus for males, uncorrected for head size

**Suppl. Figure S5:** Nomogram of total grey matter for females, uncorrected for head size

**Suppl. Figure S6:** Nomogram of total grey matter for males, uncorrected for head size

**Suppl. Figure S7:** Nomogram of total grey matter for females, corrected for head size

**Suppl. Figure S8**: Nomogram of total grey matter for males, corrected for head size

**Suppl. Figure S9:** Nomogram of hippocampus-to-total grey matter ratio for females, corrected for head size

**Suppl. Figure S10:** Nomogram of hippocampus-to-total grey matter ratio for males, corrected for head size

**Suppl. Figure S11**: Nomogram of hippocampus-to-total grey matter ratio for females, uncorrected for head size

**Suppl. Figure S12**: Nomogram of hippocampus-to-total grey matter ratio for males, uncorrected for head size

**Suppl. Figure S13:** Nomograms of head size corrected average hippocampus, calculated with sliding-window and LMS method

**Suppl. Figure S14:** Sliding-window curves with fixed age-bins for head size corrected hippocampus

**Suppl. Figure S15**: Sliding-window curves for left and right hippocampal volume across age

**Suppl. Figure S16:** Slope of hippocampal volume by age

**Suppl. Figure S17:** Sliding-window curves (Fig.8) without smoothing

**Suppl. Figure S18:** Sliding-window curves Fig.8) with smoothing kernel 10

**Suppl. Figure S19:** Sliding-window curves (Fig.8) with 20% quantile width and smoothing kernel 20

**Suppl. Figure S20**: Sliding-window curves (Fig.8) with 20% quantile width and smoothing kernel 10

**Suppl. Figure S21**: Sliding-window curves (Fig.8), uncorrected for head size

**Suppl. Figure S22:** Difference in brain volume by hypertension

**Suppl. Figure S23**: Difference in brain volume by education level

**Suppl. Figure S24**: Difference in brain volume by smoking status

**Suppl. Table 1:** *Brain volume by hypertension status and BMI correlations**

|  | **No Hypertension**  *(Mean ± std)* | **Hypertension**  *(Mean ± std)* | ***p*-value**, Effect size** | **BMI**  *(Pearson’s r, p-value**)* |
| --- | --- | --- | --- | --- |
| **Total grey matter volume** | 617,487.42 ± 26,298.42 mm^3^ | 612,994.27 ± 27938.30 mm^3^ | *p* < .001, *Hedges’ G* = .16 | *r* = -.11,  *p* < .001 |
| **Hippocampal volume** | 3868.40 ± 351.77 mm^3^ | 3846.02 ± 359.61 mm^3^ | *p* < .001, *Hedges’ G* = .07 | *r* = -.03,  *p* < .001 |
| **Parahippocampal gyrus** | 8440.67 ±  591.44 mm^3^ | 8396.09 ± 605.95 mm^3^ | *p* < .001, *Hedges’ G* = .08 | *r* = -.04,  *p* < .001 |
| **Fusiform gyrus** | 7799.46 ±  719.50 mm^3^ | 7747.10 ± 715.59 mm^3^ | *p* < .001, *Hedges’ G* = .07 | *r* = -.05,  *p* < .001 |
| **Inferior temporal gyrus** | 8431.44 ± 1033.65 mm^3^ | 8394.17 ± 1018.81 mm^3^ | *p* = .03  *Hedges’ G* = .04 | *r* = -.02,  *p* = .009 |
| **Mid temporal gyrus** | 10,744.31 ± 1110.08 mm^3^ | 10,602.11 ± 1109.54 mm^3^ | *p* < .001, *Hedges’ G* = .13 | *r* = -.04,  *p <* .001 |
| **Superior temporal gyrus** | 6364.29 ± 709.80 mm^3^ | 6297.34 ± 710.23 mm^3^ | *p* < .001, *Hedges’ G* = .09 | *r* = -.03,  *p* = .001 |
| **Temporal pole** | 9507.25 ±  892.26 mm^3^ | 9460.28 ± 918.43 mm^3^ | *p* = .002  *Hedges’ G* = .05 | *r* = -.02,  *p* = .001 |
| **N (Hippocampus)** | 15,367 | 4426 | / | / |
| ** Volumes are averaged over hemisphere and corrected for head-size and age*  *** Bonferroni-corrected* α = *.05/8 multiple comparisons = .006* | | | | |

**Suppl. Table 2:** *Brain volume by education level******

|  | **College/University**  *(Mean ± std)* | **Non-university**  *(Mean ± std)* | ***p*-value**,  Effect size** |
| --- | --- | --- | --- |
| **Total grey matter volume** | 616,558.75 ± 26,825.60 mm^3^ | 616,444.71 ± 26,528.94 mm^3^ | *p* = .77 |
| **Hippocampal volume** | 3872.48 ± 358.24 mm^3^ | 3848.42 ± 345.29 mm^3^ | *p* < .001,  *Hedges’ G* = .07 |
| **Parahippocampal gyrus** | 8436.36 ± 596.68 mm^3^ | 8422.77 ± 591.79 mm^3^ | *p* = .12 |
| **Fusiform gyrus** | 7801.96 ± 729.04 mm^3^ | 7766.99 ± 701.35 mm^3^ | *p* = .001,   *Hedges’ G* = .05 |
| **Inferior temporal gyrus** | 8433.30 ± 1040.77 mm^3^ | 8410.89 ± 1012.63 mm^3^ | *p* = .14 |
| **Mid temporal gyrus** | 10,705.05 ± 1118.75 mm^3^ | 10,728.99 ± 1109.93 mm^3^ | *p* = .14 |
| **Superior temporal gyrus** | 6350.49 ± 717.46 mm^3^ | 6349.16 ± 701.52 mm^3^ | *p* = .89 |
| **Temporal pole** | 9514.02 ± 906.29 mm^3^ | 9468.41 ± 884.33 mm^3^ | *p* < 001,  *Hedges’ G* = .05 |
| **N (Hippocampus)** | 12275 | 7371 | / |
| ** Volumes are averaged over hemisphere and corrected for head-size and age*  *** Bonferroni-corrected* α = *.05/8 multiple comparisons = .006* | | | |

**Suppl. Table 3:** *Brain volume by smoking status**

|  | **Never Smoked**  *(Mean ± std)* | **Previous Smoker**  *(Mean ± std)* | **Current Smoker** *(Mean ± std)* | ***F-test***** |
| --- | --- | --- | --- | --- |
| **Total grey matter volume** | 618,402.92 ± 26,445.67 mm^3^ | 613,808.99 ± 26,817.75 mm^3^ | 609,778.46 ± 27,280.52 mm^3^ | *F(3,19779) = 60.12, p* < .001 |
| **Hippocampal volume** | 3871.85 ± 353.02 mm^3^ | 3853.36 ± 353.75 mm^3^ | 3819.70 ± 353.53 mm^3^ | *F(3,19541) = 8.26, p* < .001 |
| **Parahippocampal gyrus** | 8444.84 ± 592.86 mm^3^ | 8414.13 ± 594.62 mm^3^ | 8359.77 ± 617.07 mm^3^ | *F(3,19660) = 7.65, p* < .001 |
| **Fusiform gyrus** | 7808.68 ± 717.71 mm^3^ | 7759.14 ± 719.81 mm^3^ | 7732.03 ± 720.93 mm^3^ | *F(3,19660) = 8.62, p* < .001, |
| **Inferior temporal gyrus** | 8445.65 ± 1030.63 mm^3^ | 8402.03 ± 1025.52 mm^3^ | 8297.72 ± 1051.02 mm^3^ | *F(3,19568) = 6.81, p* < .001 |
| **Mid temporal gyrus** | 107,46.47 ± 1109.89 mm^3^ | 10,668.04 ± 1104.04 mm^3^ | 10,583.20 ± 1185.32 mm^3^ | *F(3,19609) = 10.98, p* < .001 |
| **Superior temporal gyrus** | 6371.91 ± 714.98 mm^3^ | 6317.67 ± 702.42 mm^3^ | 6279.76 ± 705.16 mm^3^ | *F(3,19616) = 10.91,  p* < .001 |
| **Temporal pole** | 9527.57 ± 899.46 mm^3^ | 9453.23 ± 891.44 mm^3^ | 9382.81 ± 923.98 mm^3^ | *F(3,19719) = 14.11, p* < .001 |
| **N (Hippocampus)** | 122232 | 6597 | 759 | / |
| ** Volumes are averaged over hemisphere and corrected for head-size and age*  ******** *Bonferroni-corrected* α = *.05/8 multiple comparisons = .006* | | | | |

**A**

**B**

|  | **Never Smoked  VS  Previous Smoker** | **Previous Smoker  VS  Current Smoker** | **Never smoked  VS  Current Smoker** |
| --- | --- | --- | --- |
| **Total grey matter volume** | **🡩** | **🡩** | **🡩** |
| **Hippocampal volume** | **🡩** | **🡩** | **🡩** |
| **Parahippocampal gyrus** | No difference | **🡩** | **🡩** |
| **Fusiform gyrus** | **🡩** | No difference | **🡩** |
| **Inferior temporal gyrus** | No difference | **🡩** | **🡩** |
| **Mid temporal gyrus** | **🡩** | No difference | **🡩** |
| **Superior temporal gyrus** | **🡩** | No difference | **🡩** |
| **Temporal pole** | **🡩** | No difference | **🡩** |

**🡩 = significantly larger volume**

**Suppl. Table 4:** *Brain volume by hemisphere*

|  | **Left hemisphere**  *(Mean ± std)* | **Right hemisphere**  *(Mean ± std)* | ***p*-value*,  Effect size** |
| --- | --- | --- | --- |
| **Hippocampal volume** | 3807.70 ± 451.63 mm^3^ | 3915.91 ± 464.43 mm^3^ | *p* < .001,  *Hedges’ G* = .24 |
| **Parahippocampal gyrus** | 4642.01 ± 537.72 mm^3^ | 4383.59 ± 551.59 mm^3^ | *p* < .001,  *Hedges’ G* = .48 |
| **Fusiform gyrus** | 5477.83 ± 713.48 mm^3^ | 4621.58 ± 612.06 mm^3^ | *p* < .001,  *Hedges’ G* = 1.30 |
| **Inferior temporal gyrus** | 5580.50 ± 942.11 mm^3^ | 5689.91 ± 904.90 mm^3^ | *p* < .001,  *Hedges’ G* = .12 |
| **Middle temporal gyrus** | 7155.30 ± 1076.31 mm^3^ | 7121.20 ± 1017.23 mm^3^ | *p* = .61 |
| **Superior temporal gyrus** | 4055.07 ± 647.87 mm^3^ | 4591.15 ± 694.65 mm^3^ | *p* < .001,  *Hedges’ G* = .80 |
| **Temporal pole** | 9523.30 ± 1260.67 mm^3^ | 9473.84 ± 1213.86 mm^3^ | *p* <.001,  *Hedges’ G* = .04 |

** Bonferroni-corrected* α = *.05/7 multiple comparisons = .007*

**Suppl. Table 5:** *Brain volume by sex*

|  | **Male**  *(Mean ± std)* | **Female**  *(Mean ± std)* | ***p*-value**,  Effect size** |
| --- | --- | --- | --- |
| **Total grey matter volume** | 614,423.67 ± 28,265.18 mm^3^ | 618,328.53 ± 25,151.14 mm^3^ | *p* < .001,  *Hedges’ G* = .15 |
| **Hippocampal volume** | 3866.54 ± 383.33  mm^3^ | 3860.62 ± 325.01 mm^3^ | *p* = .24 |
| **Parahippocampal gyrus** | 8499.62 ± 619.13  mm^3^ | 8369.57 ± 565.75  mm^3^ | *p* < .001,  *Hedges’ G* = .22 |
| **Fusiform gyrus** | 7825.78 ± 749.22  mm^3^ | 7754.93 ± 689.34  mm^3^ | *p* < .001,  *Hedges’ G* = .10 |
| **Inferior temporal gyrus** | 8454.76 ± 1076.24  mm^3^ | 8395.22 ± 987.53  mm^3^ | *p* < .001,  *Hedges’ G* = .06 |
| **Middle temporal gyrus** | 10,643.07 ± 1152.80 mm^3^ | 10,774.03 ± 1069.91 mm^3^ | *p* < .001,  *Hedges’ G* = .12 |
| **Superior temporal gyrus** | 6331.83 ± 732.669  mm^3^ | 6364.79 ± 689.78  mm^3^ | *p* = .001,  *Hedges’ G* = .05 |
| **Temporal pole** | 9571.71 ± 941.40 mm^3^ | 9429.92 ± 852.70  mm^3^ | *p* < .001,  *Hedges’ G* = .16 |

** Volumes are averaged over hemisphere and corrected for head-size and age*

******** *Bonferroni-corrected* α = *.05/8 multiple comparisons = .006*

**Suppl. Table 6:** *Model Estimates from joinpoint regression of total grey matter*

|  | **Female** | **Male** |
| --- | --- | --- |
| **DF** | 21 | 21 |
| **Joinpoint** | 56-57 years | 62-63 years |
| **Joinpoint 95% LCL** | 53-54 years | 57-58 years |
| **Joinpoint 95% UCL** | 58-59 years | 66-67 years |
| **Slope Change Estimate** | -1273.98 mm^3^ | -754.14 mm^3^ |
| **Slope Change Std Error** | 427.19 mm^3^ | 154.85 mm^3^ |
| **Slope Change Test Statistic** | -2.98 | -4.87 |
| **Slope Change  p-value** | .007 | < .0001 |


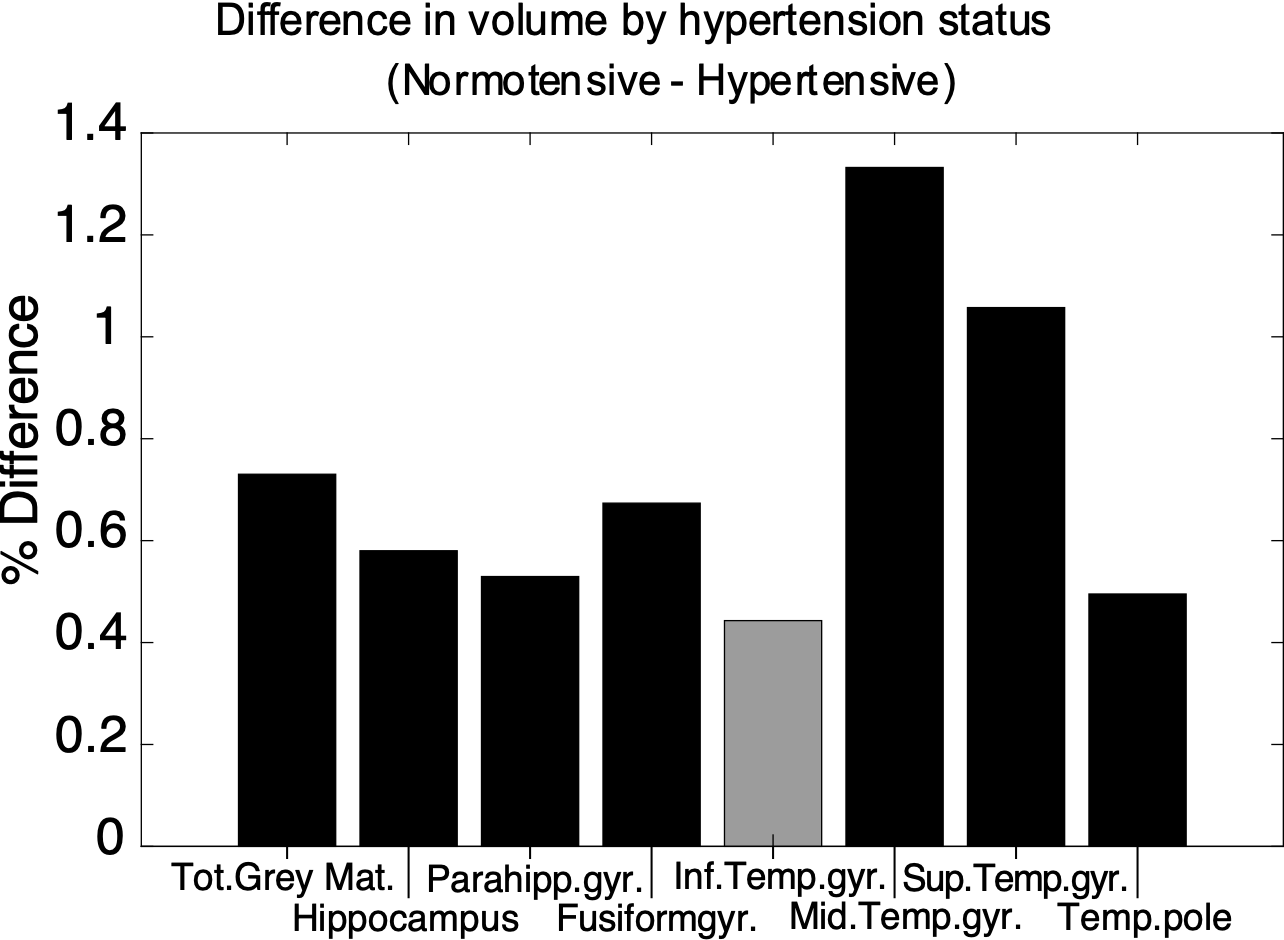


**Suppl. Figure 1:** *Difference in brain volume by hypertension status*

Percent difference in volumes was calculated with $\frac{\left| normotensive-hypertensive \right|}{\frac{normotensive+hypertensive}{2}}*100$. Positive percent differences correspond to larger volumes in participants without hypertension. Black bars indicate a significant corresponding t-test for volume differences at *p < .005 (Bonferroni-corrected* α = *.05/8 = .006)*


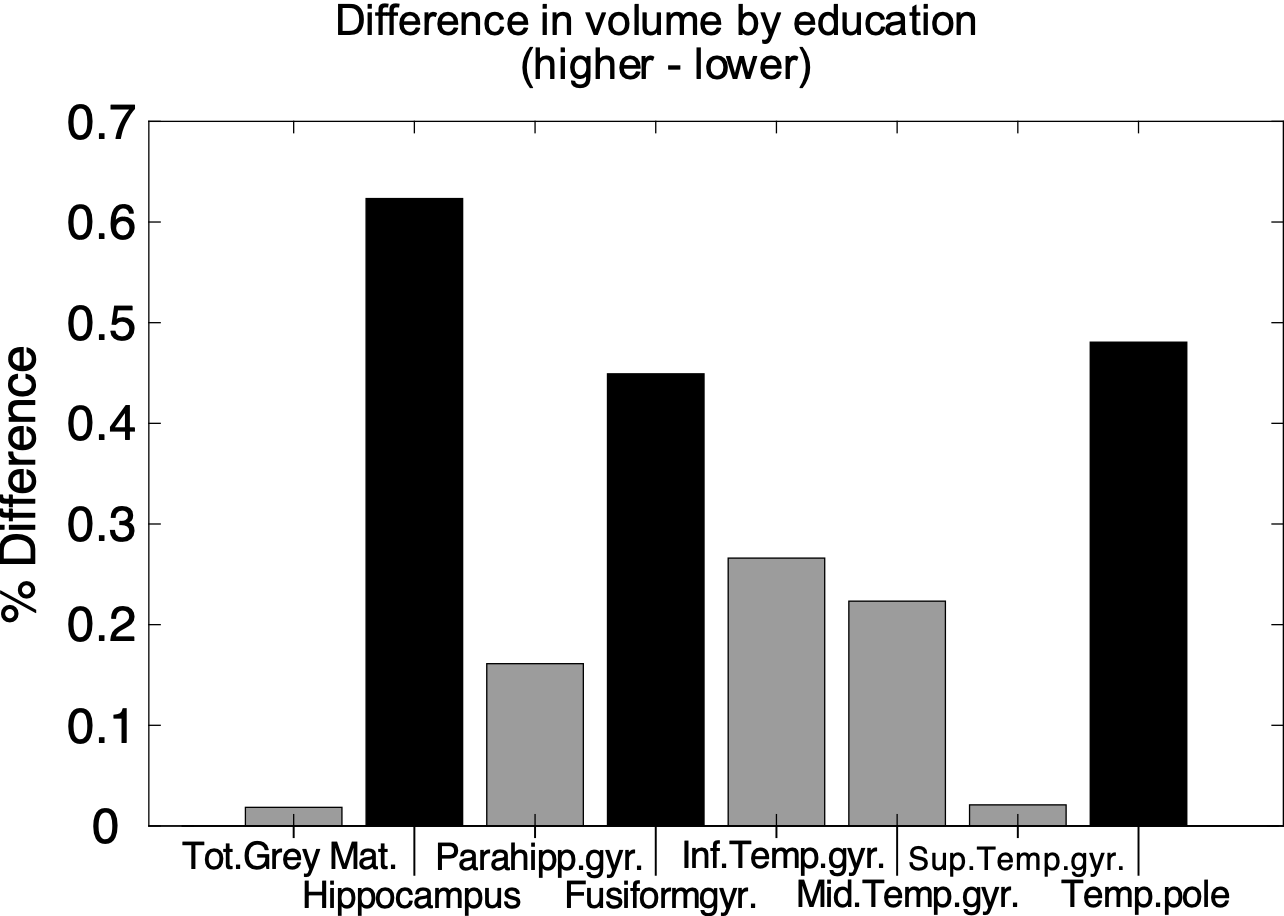


**Suppl. Figure 2:** *Percent volume differences between participants with lower and higher education levels*

Percent difference in volumes was calculated with $\frac{\left| higher-lower \right|}{\frac{higher+lower}{2}}*100$. Positive percent differences correspond to larger volumes in participants with higher education levels. Black bars indicate a significant corresponding t-test for volume differences at *p < .001 (Bonferroni-corrected* α = *.05/8 = .006).*

**
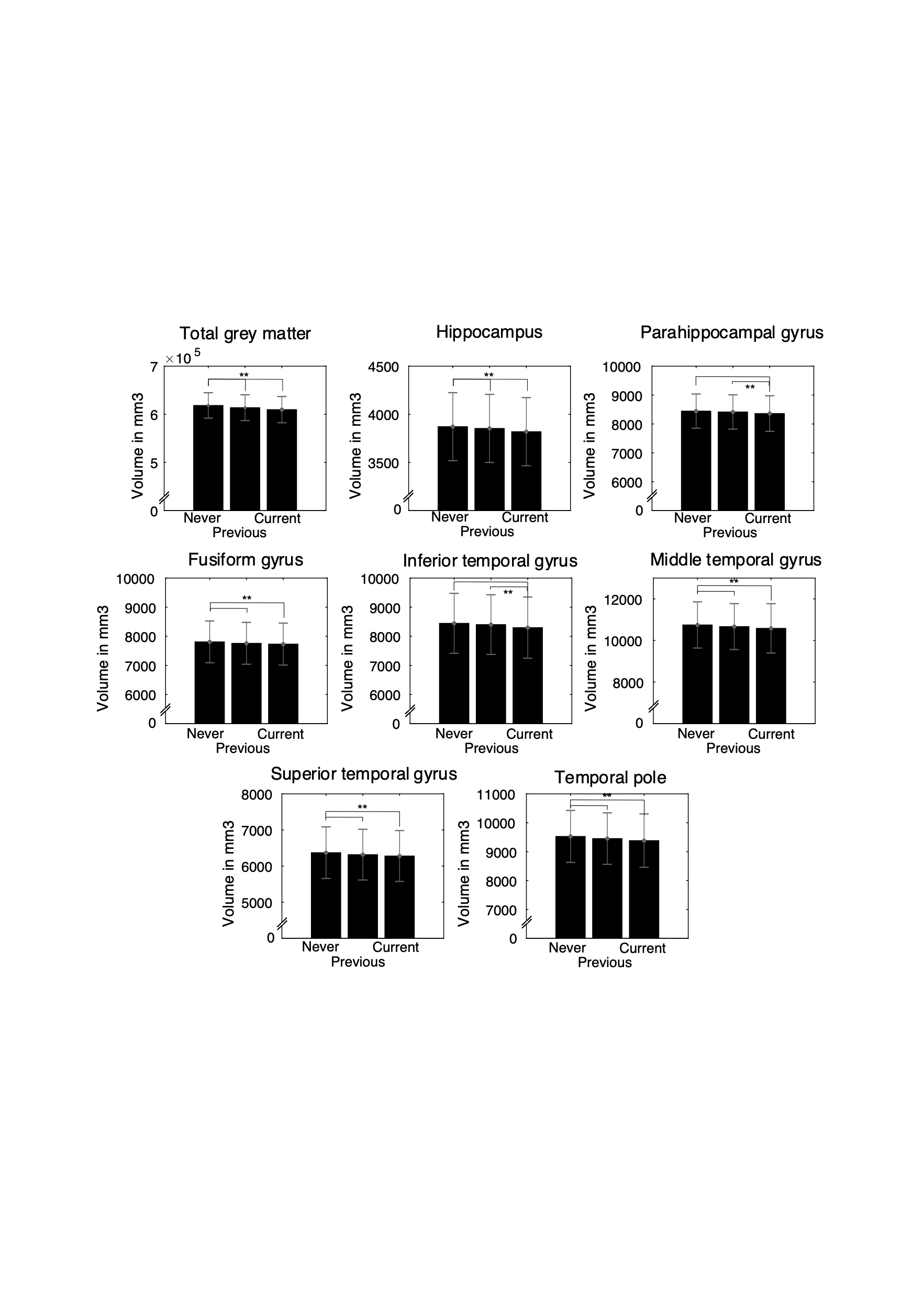
**

**Suppl. Figure 3*:*** *Bar graphs for brain volume by smoking status.*

Participants indicated whether they are current smokers, previous smokers, or whether they have never smoked. Error bars show standard deviation.

**significant at *p < .001 (Bonferroni-corrected* α = *.05/8 = .006)*


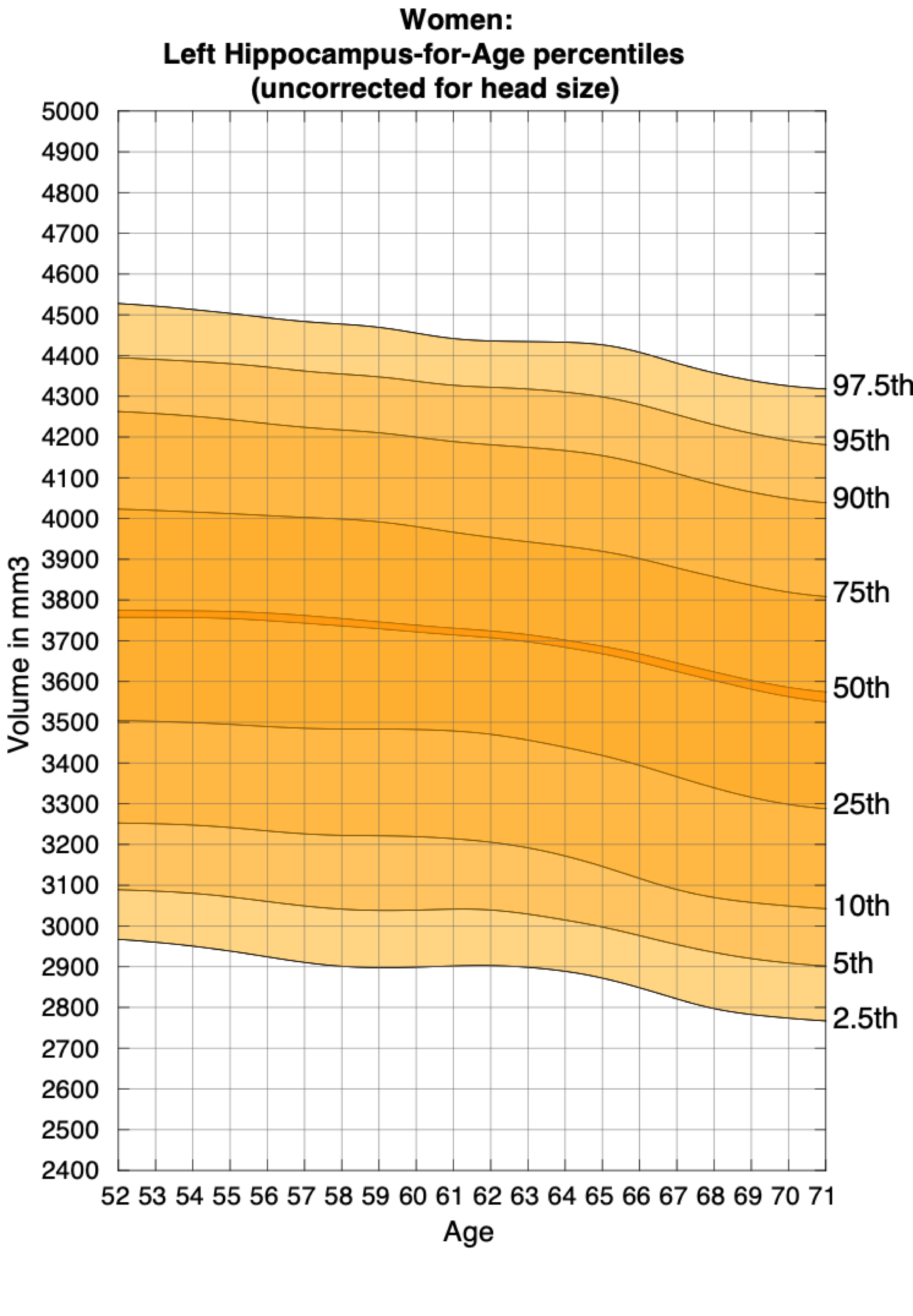


**Suppl. Figure S4**: *Nomogram of head size un-corrected left hippocampus for females*


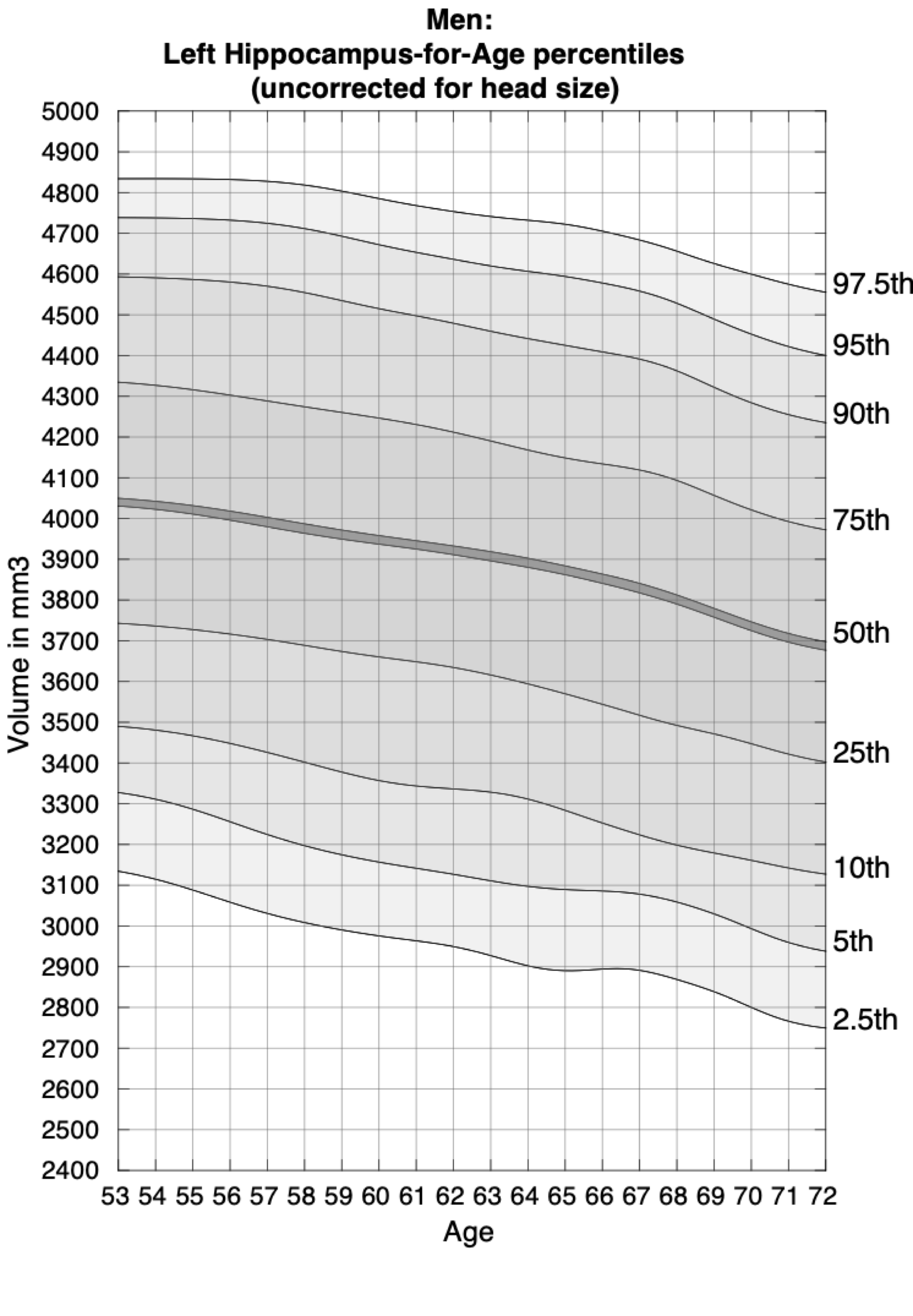


**Suppl. Figure S5**: *Nomogram of head size u-ncorrected left hippocampus for males*

**
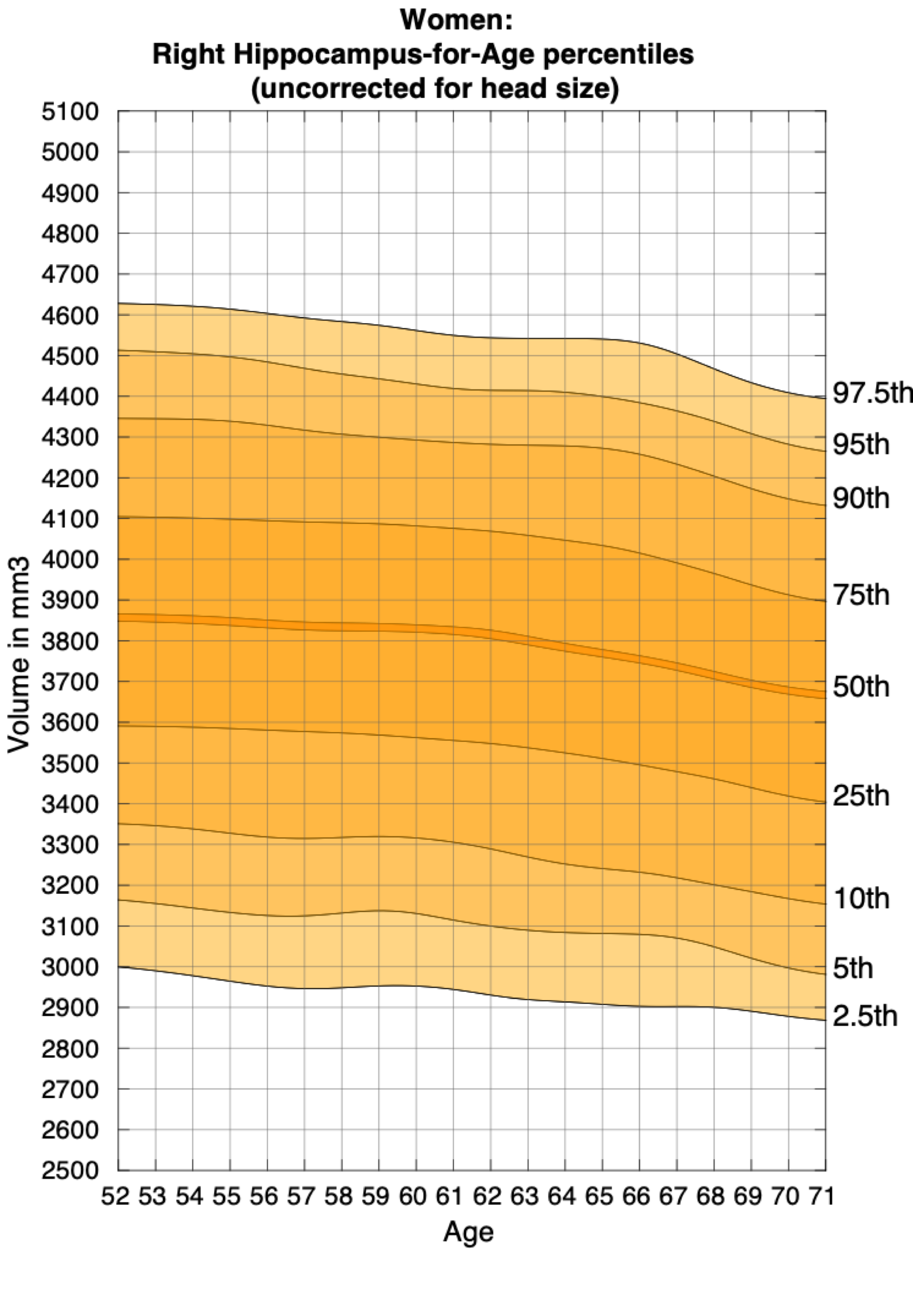
Suppl. Figure S6*:*** *Nomogram of head size un-corrected right hippocampus for females*


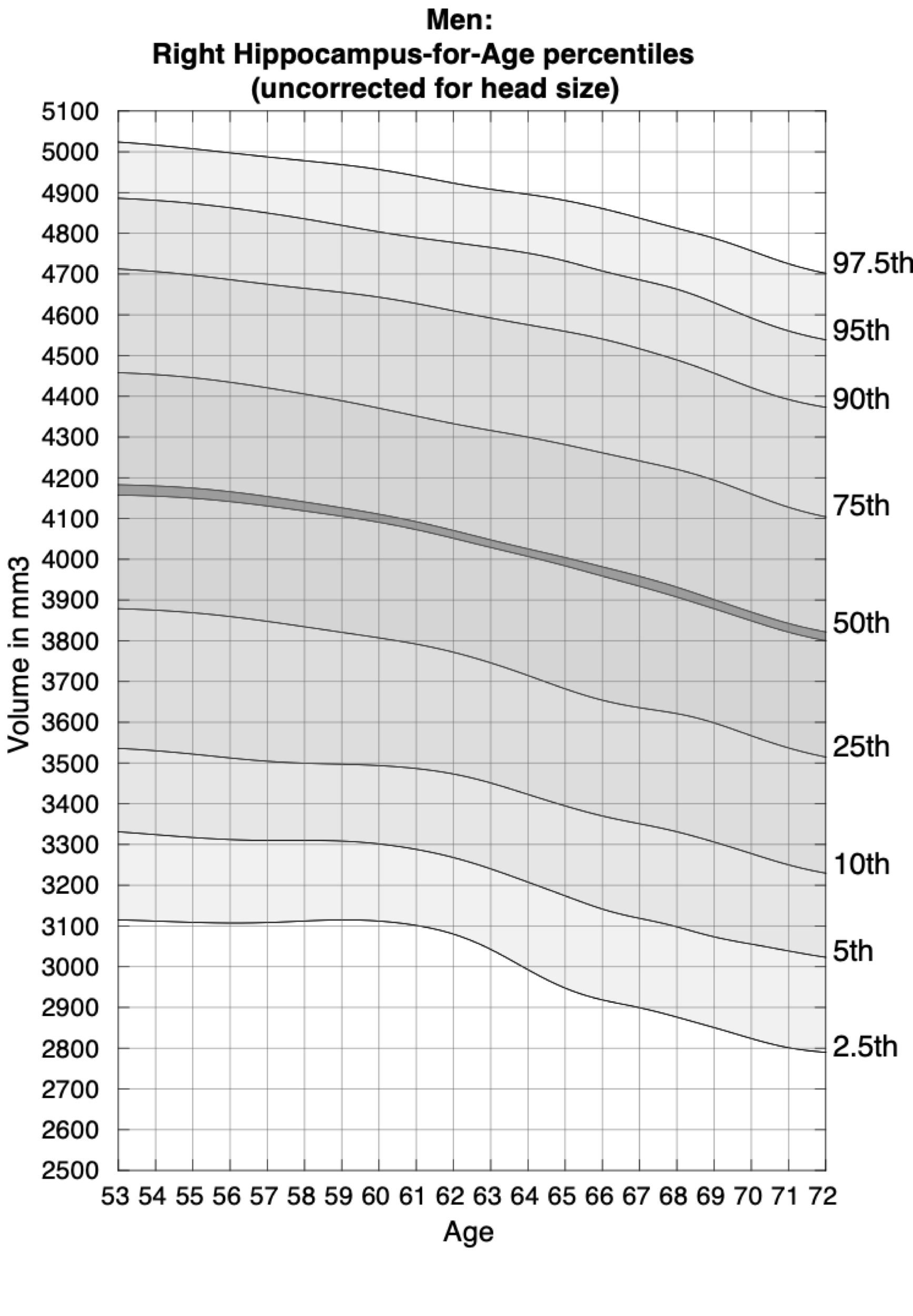


**Suppl. Figure S7***: Nomogram of head size un-corrected right hippocampus for males*


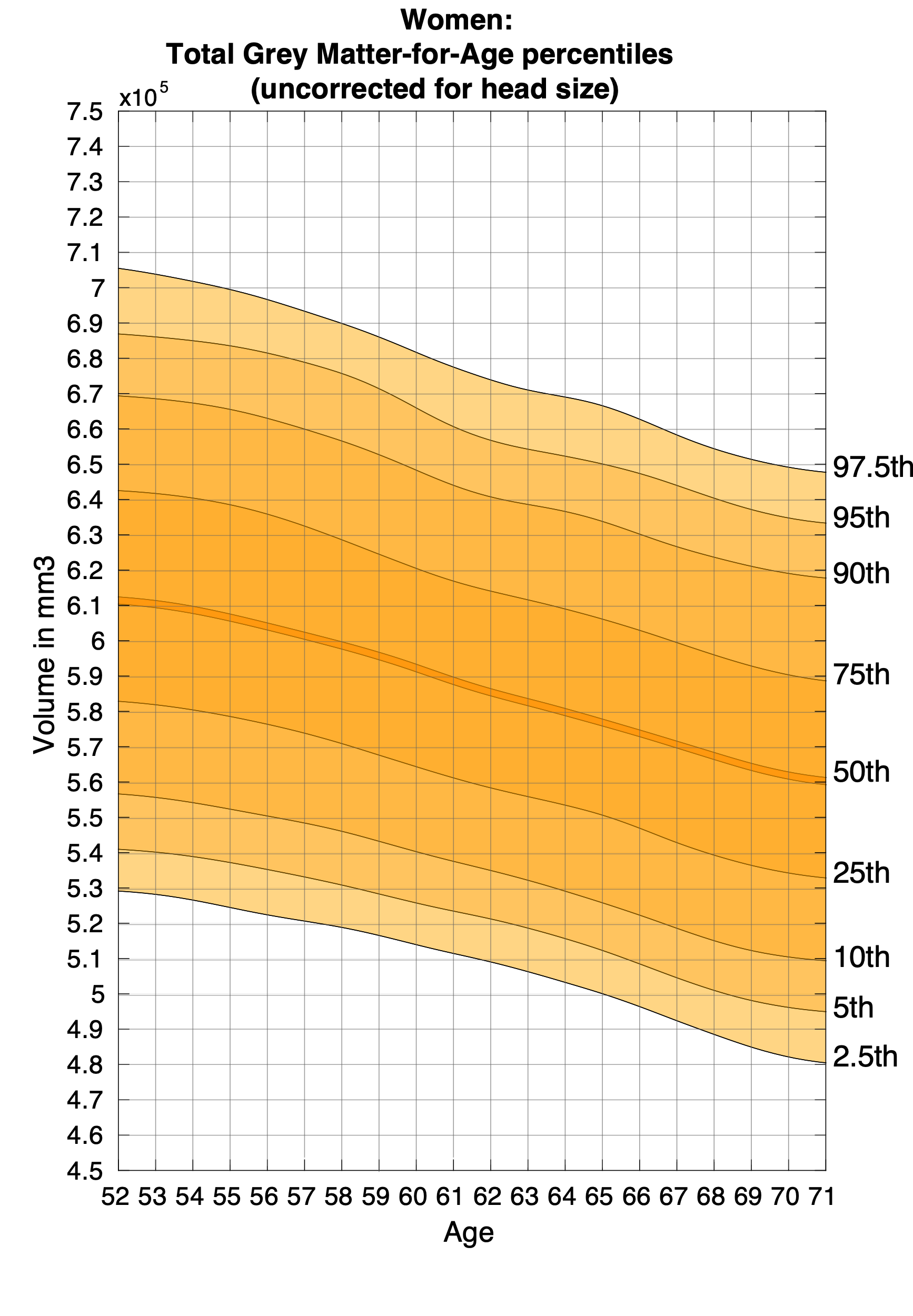


**Suppl. Figure S8:** *Nomogram of head size un-corrected total grey matter for females*

**
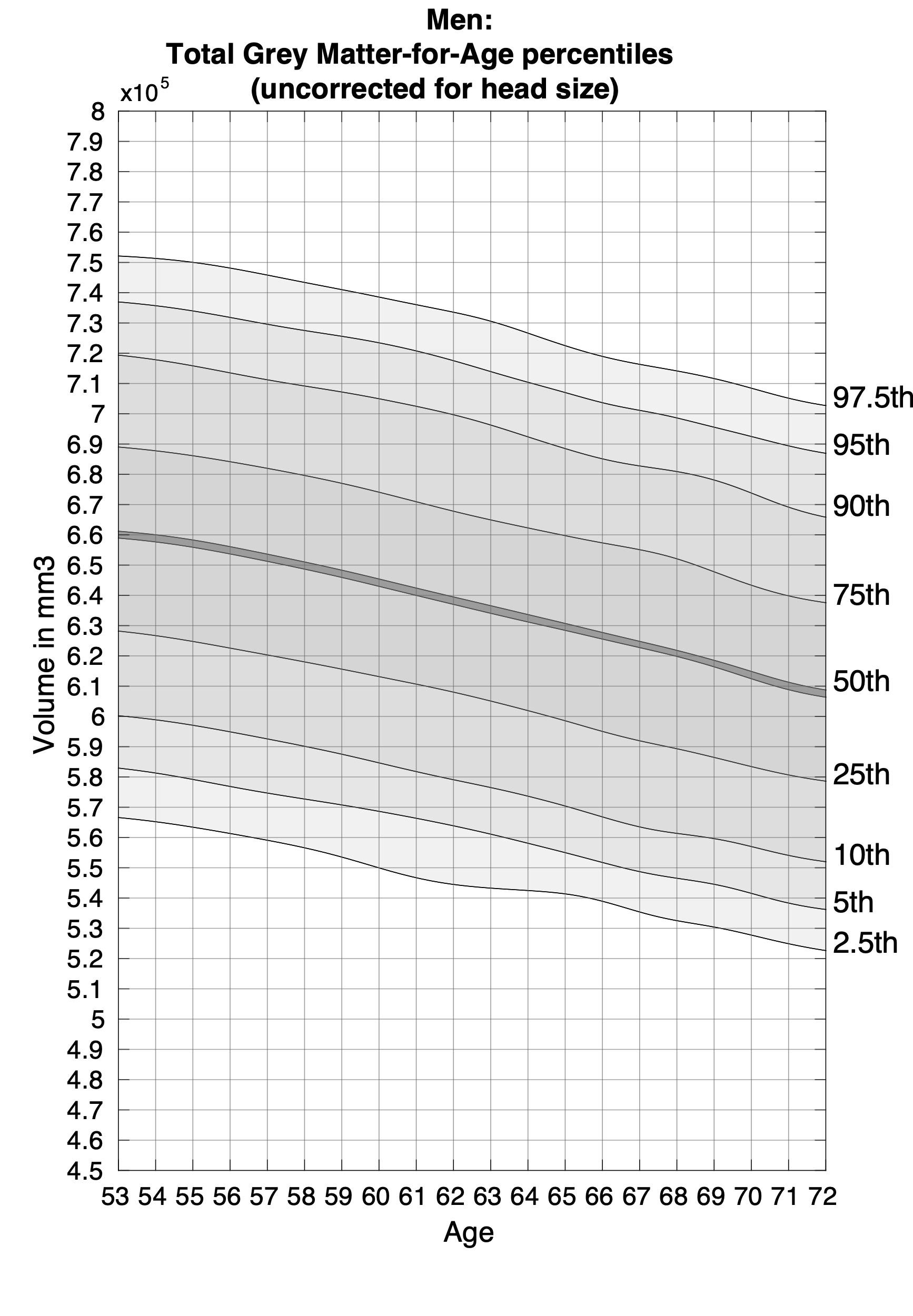
 Suppl. Figure S9*:*** *Nomogram of head size un-corrected total grey matter for males*

**
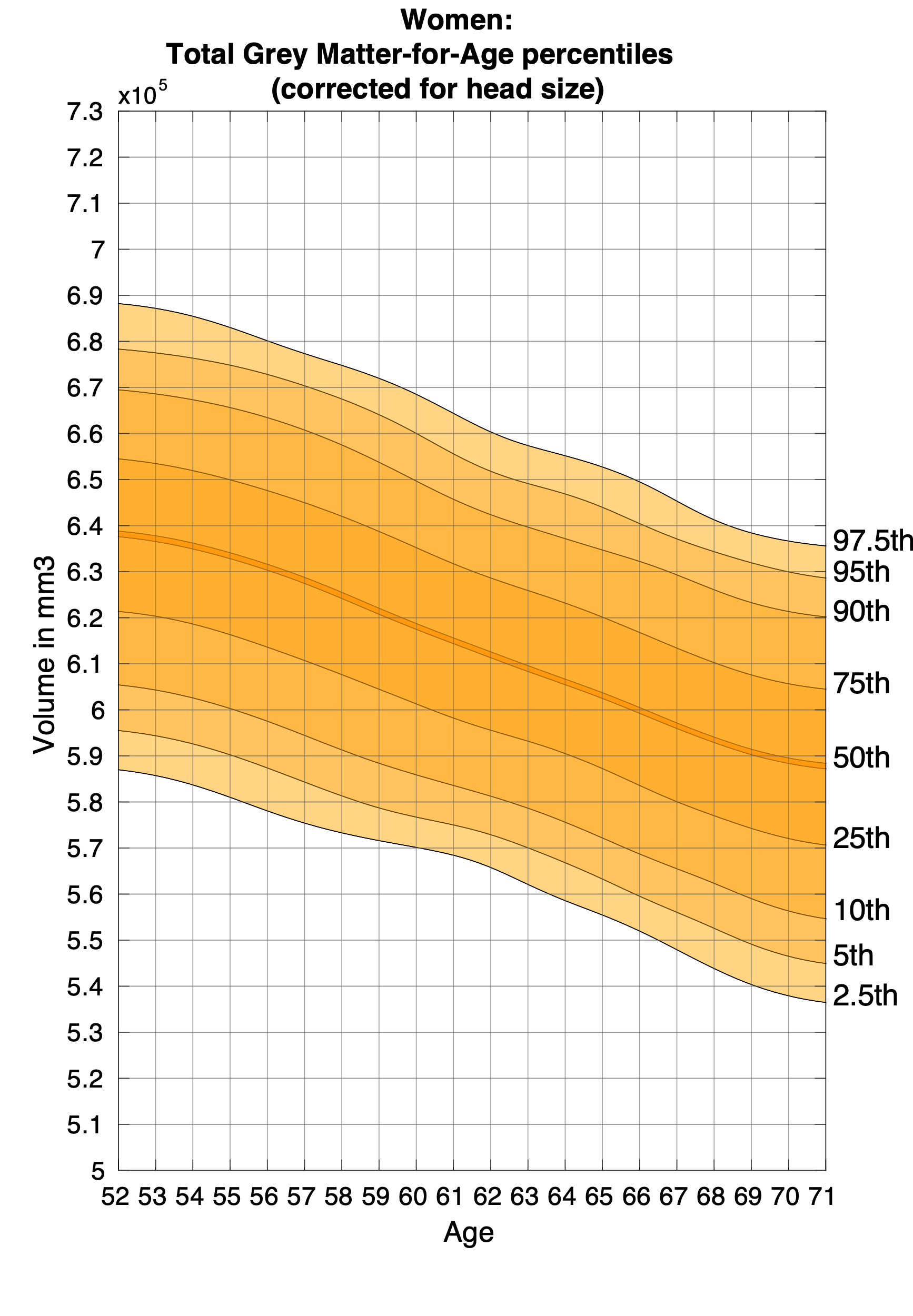
Suppl. Figure S10:** *Nomogram of head size corrected total grey matter for females*

**
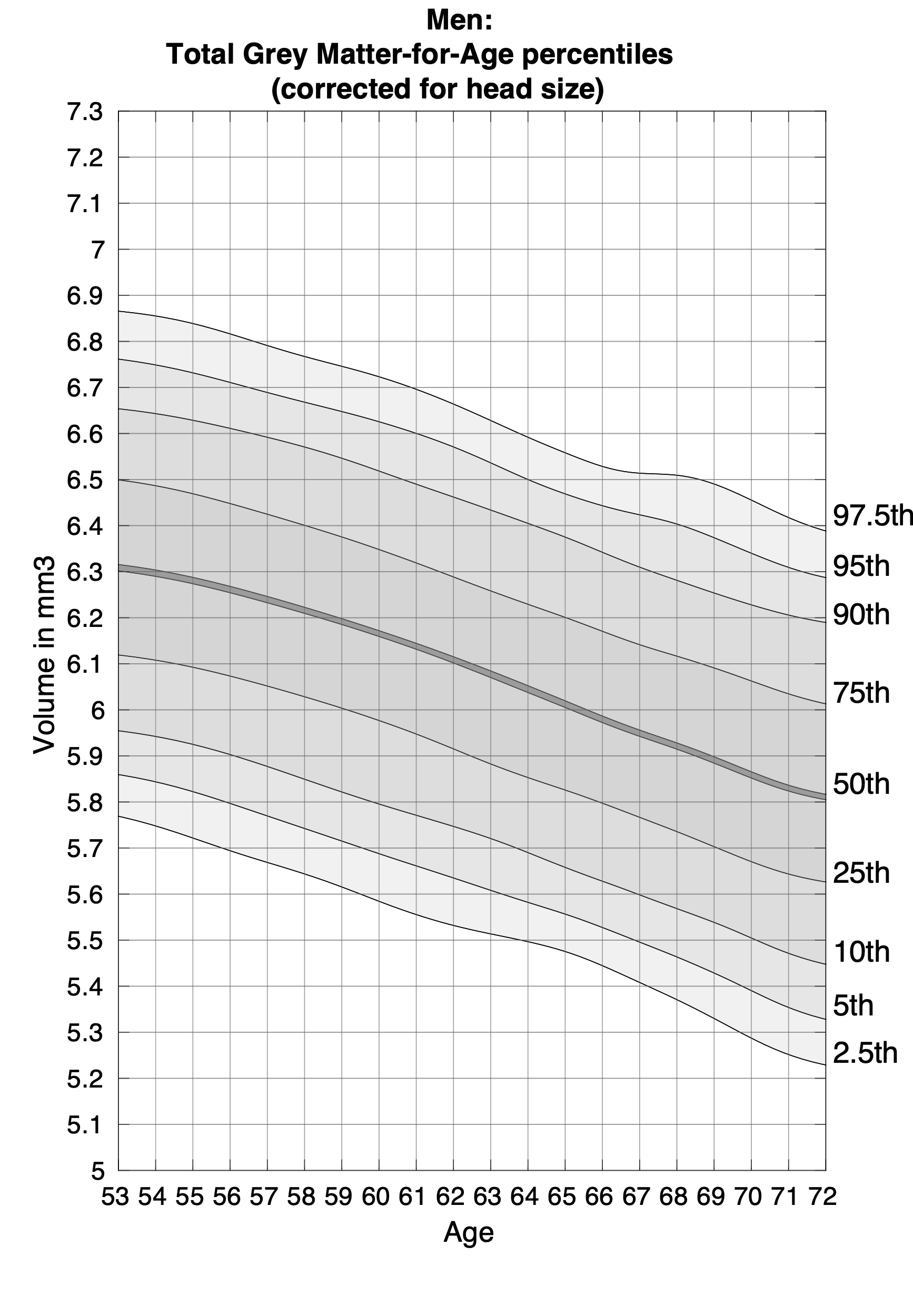
Suppl. Figure S11**: *Nomogram of head size corrected total grey matter for males*


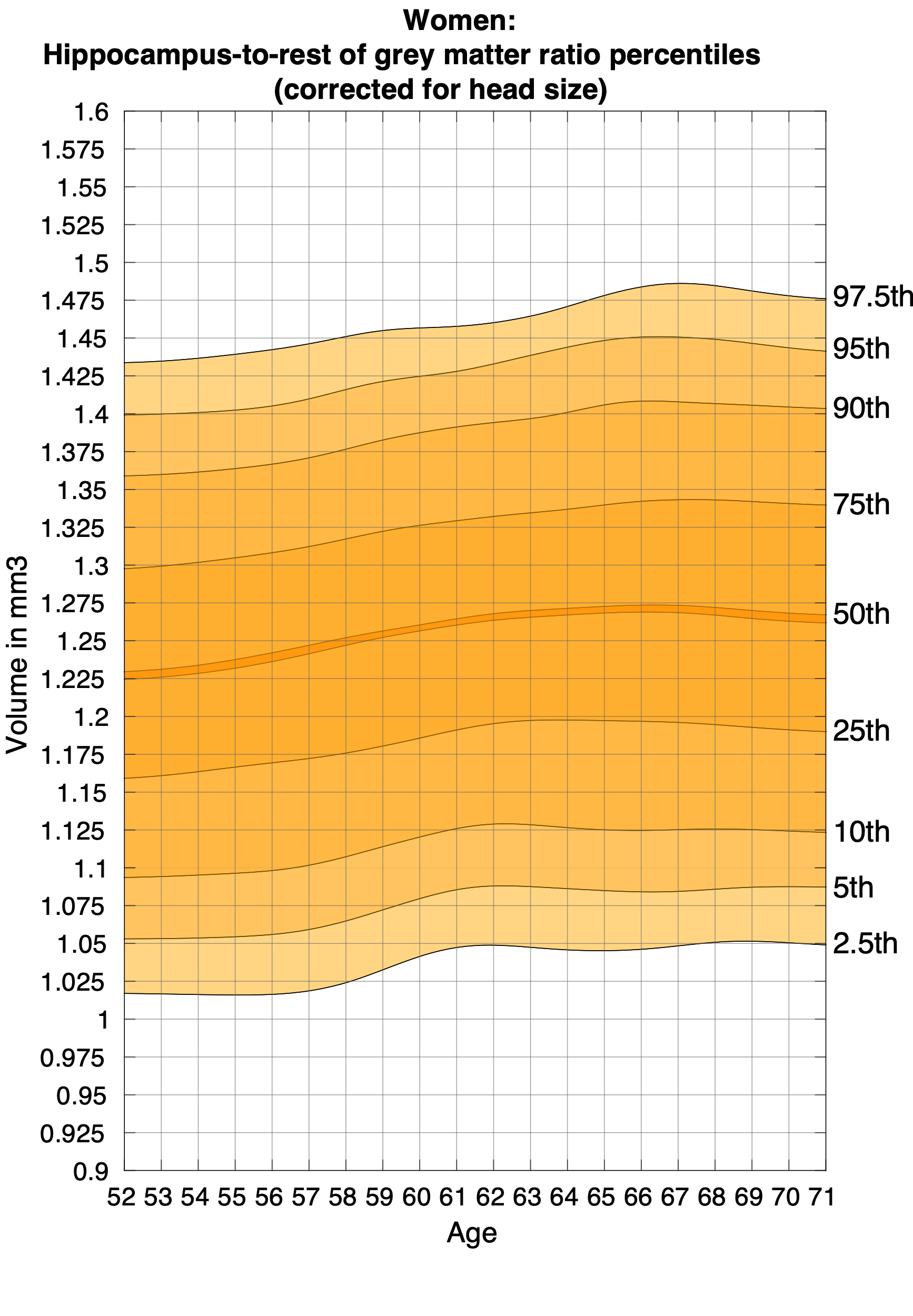


**Suppl. Figure S12**: *Nomogram of head size corrected hippocampus – to – rest of total grey matter ratio for females*


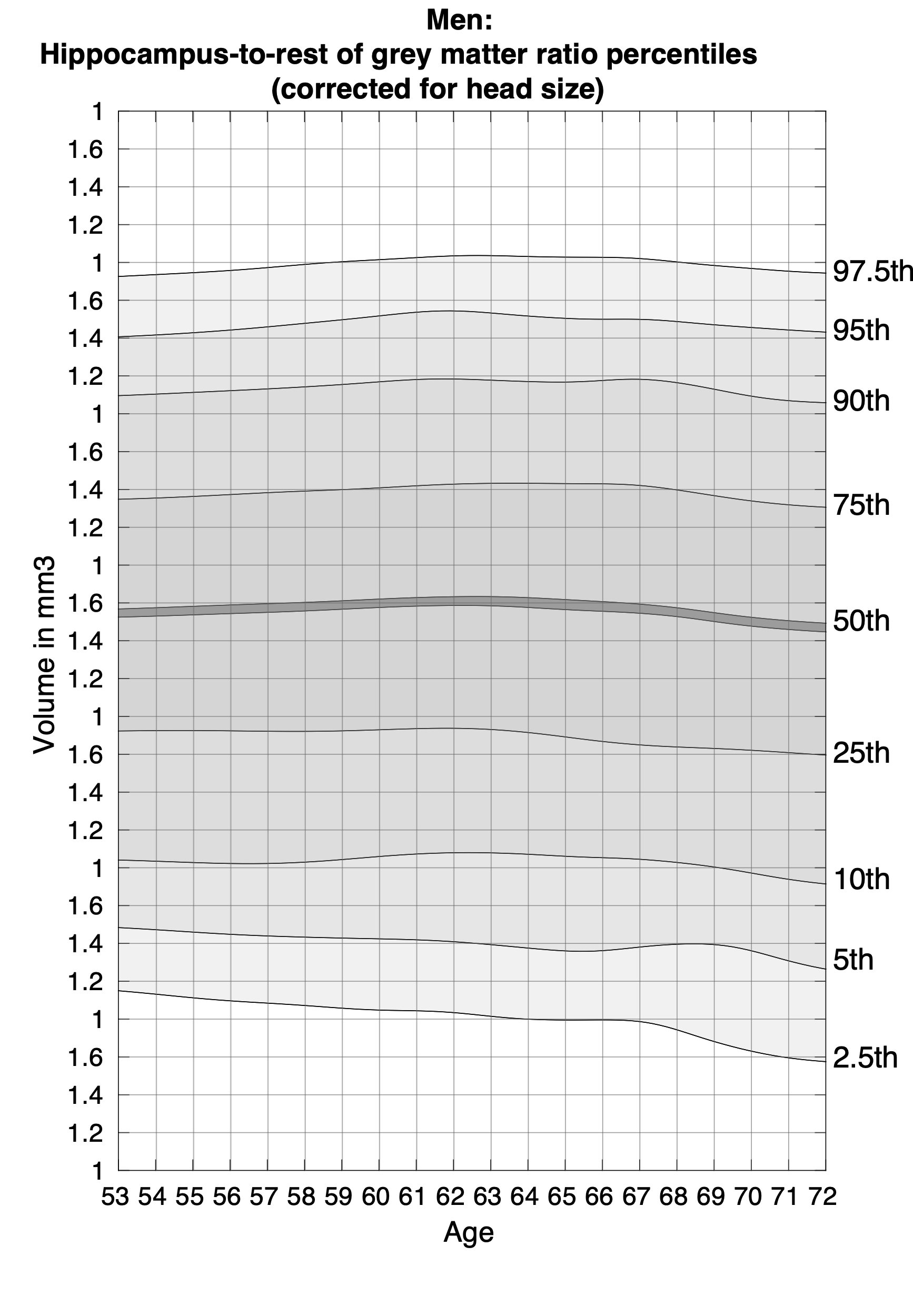


**Suppl. Figure S13**: *Nomogram of head size corrected hippocampus – to – rest of total grey matter ratio for males*


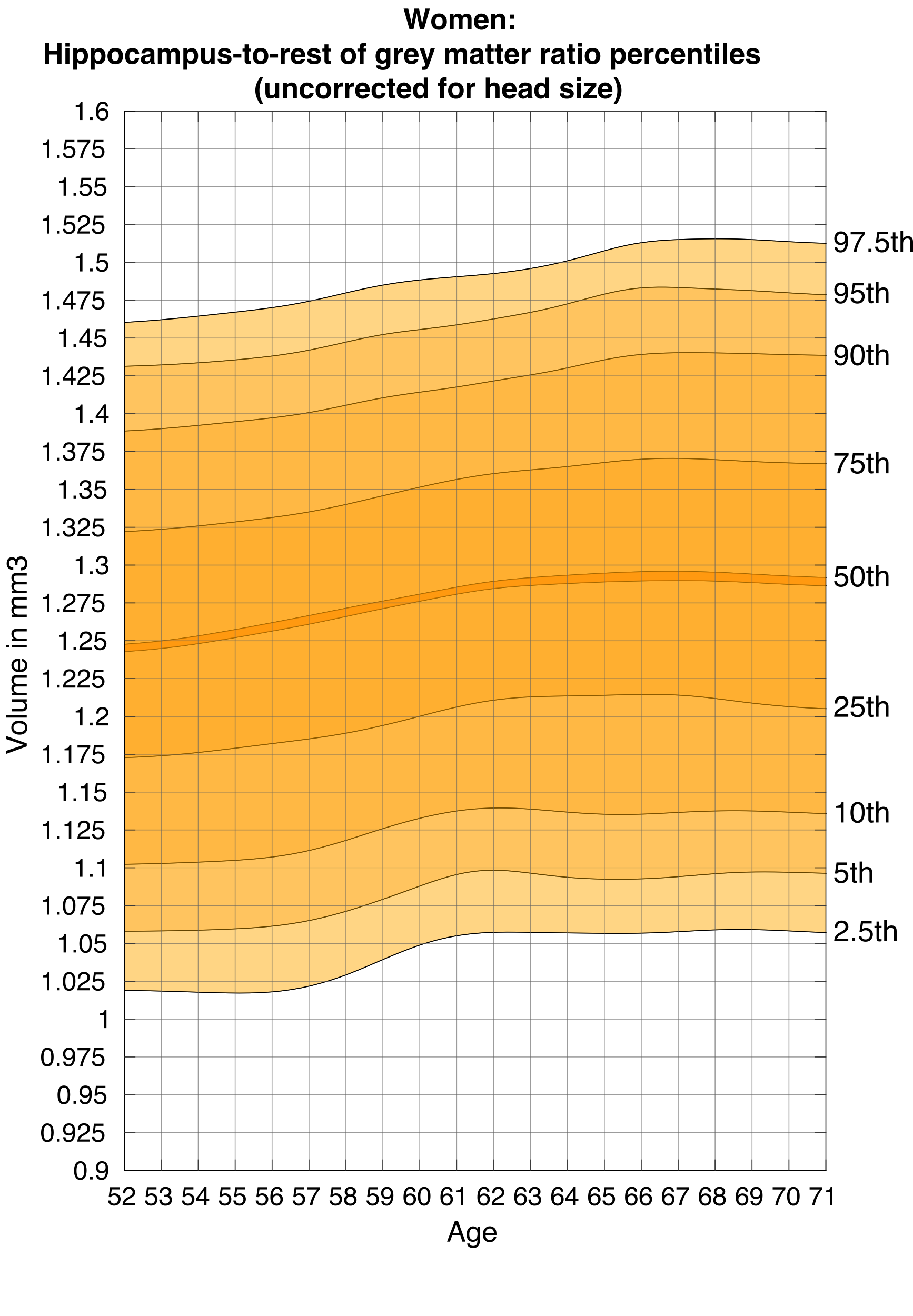


**Suppl. Figure S14**: *Nomogram of head size un-corrected hippocampus – to – rest of total grey matter ratio for females*


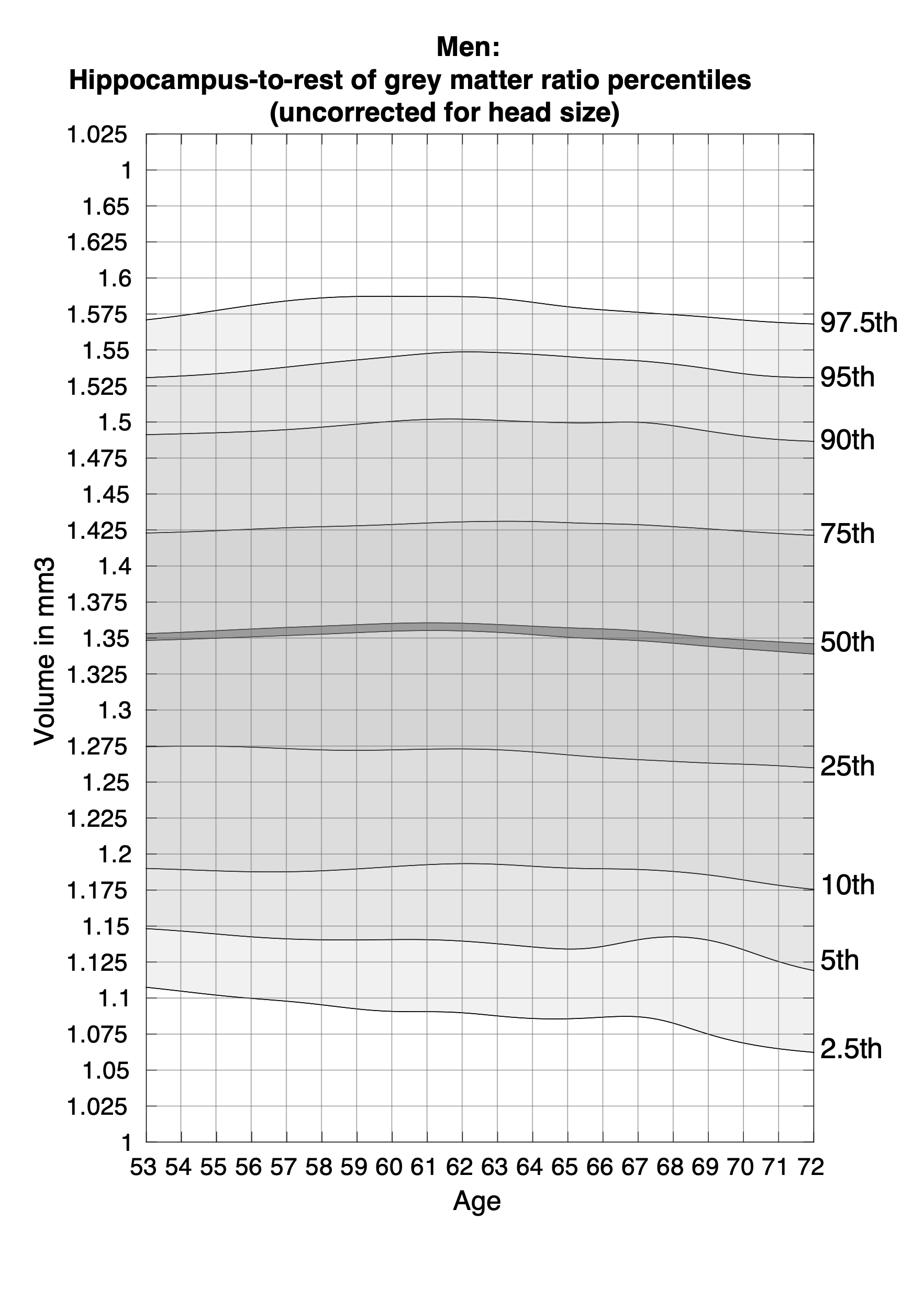


**Suppl. Figure S15**: *Nomogram of head size un-corrected hippocampus – to – rest of total grey matter ratio for males*


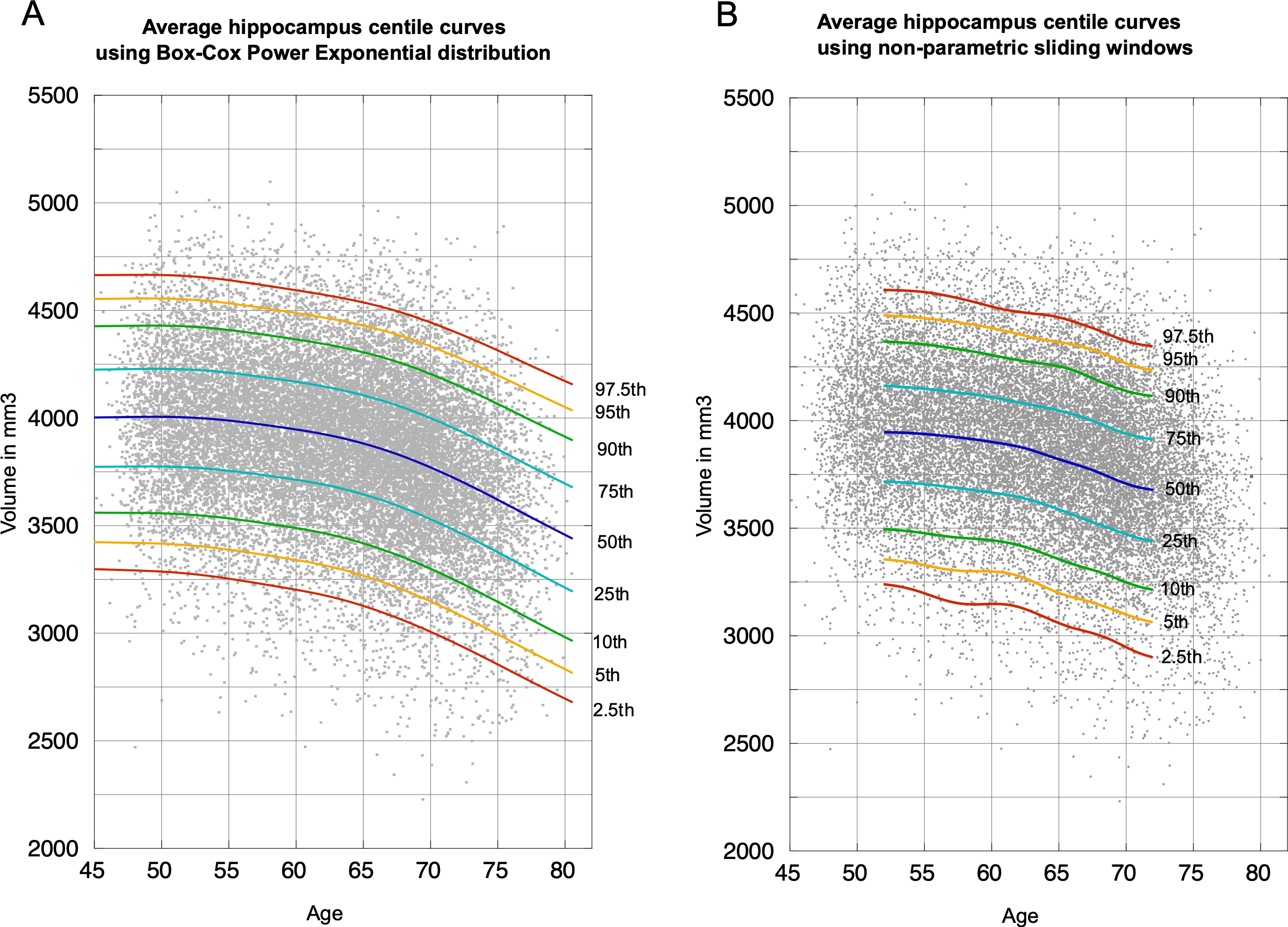


**Suppl. Figure S16:** *Nomograms of head size corrected average hippocampus, estimated with sliding-window method and Box-Cox Power Exponential distribution*

**A**. Average hippocampus centile curves estimated using Box-Cox Power Exponential (BCPE) distribution, and plotted over raw data. Average hippocampal volume has been corrected for head size and is analysed for men and women combined. The model was fitted using the *lms()* function (Stasinopoulos et al. 2007) in *RStudio* version 1.1.383. The function selects the best distribution according to Generalized Akaike Information Criterion with penalty k = 2, in this case BCPE. **B**. Average hippocampus centile curves using non-parametric sliding window analysis, corrected for head size and plotted over raw data.


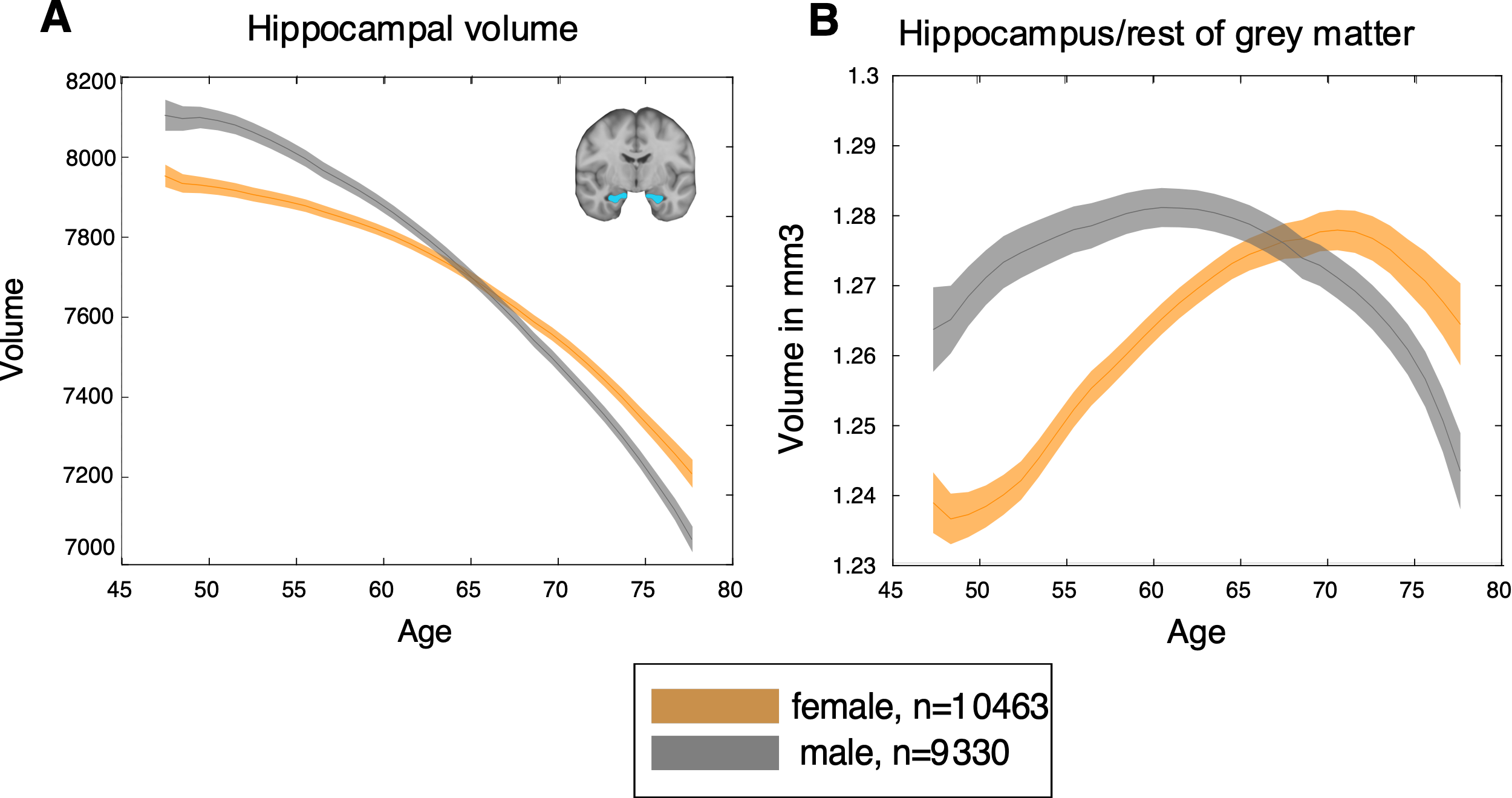


**Suppl. Figure S17:** *Sliding-window curves with fixed age-bins for head size corrected hippocampus*

**A**. Mean bilateral hippocampal volume including standard errors as a function of age, corrected for head size. **B**. Mean hippocampal volume to rest of grey matter ratio including standard errors as a function of age.


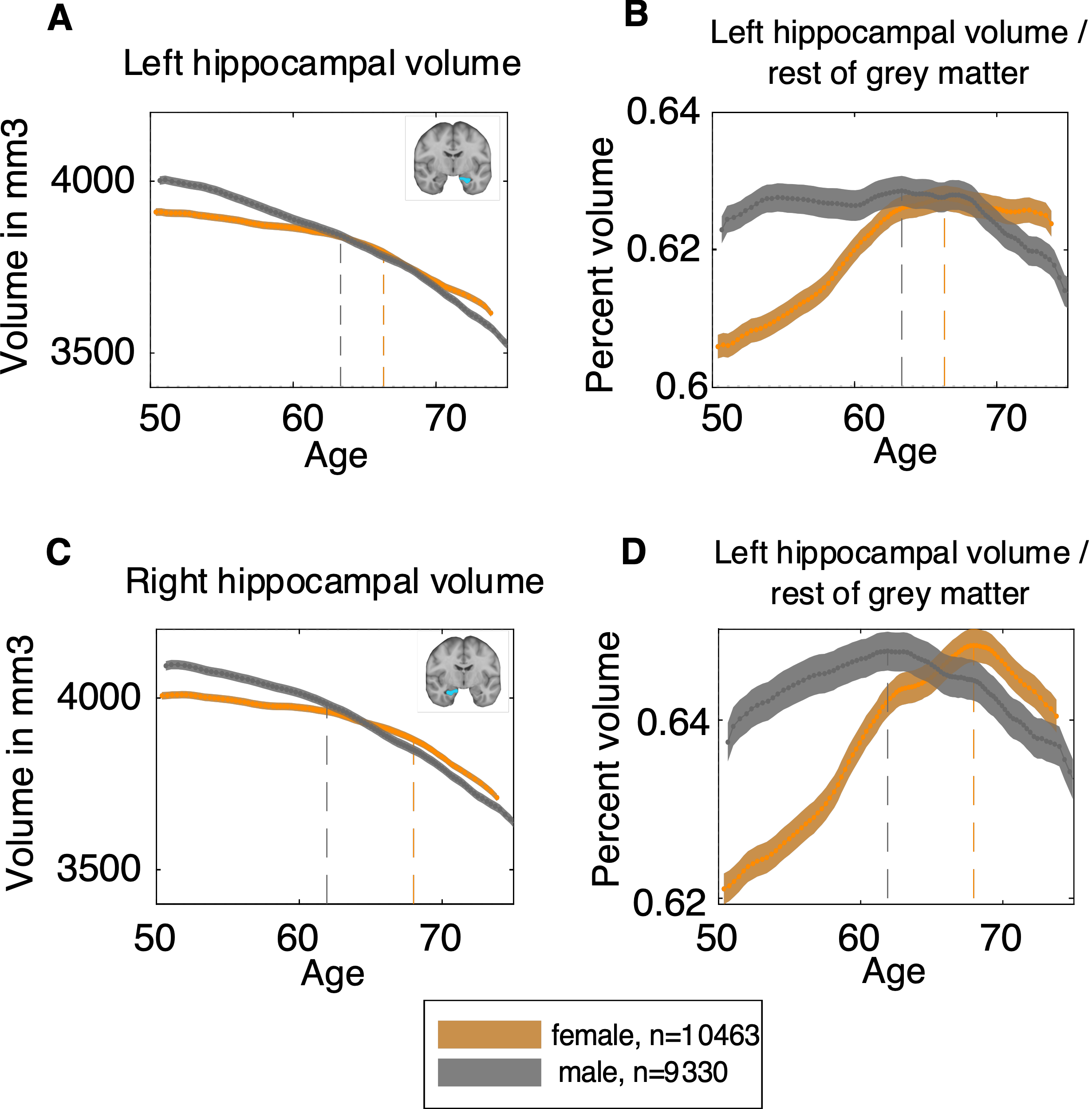


**Suppl. Figure S18**: *Sliding-window curves of left and right hippocampus across age*

**A**. Mean left hippocampal volume including standard errors as a function of age, corrected for head size. **B**. Mean left hippocampal volume to rest of grey matter ratio including standard errors as a function of age. **C**. Mean right hippocampal volume including standard errors as a function of age, corrected for head size. **D**. Mean right hippocampal volume to rest of grey matter ratio including standard errors as a function of age.


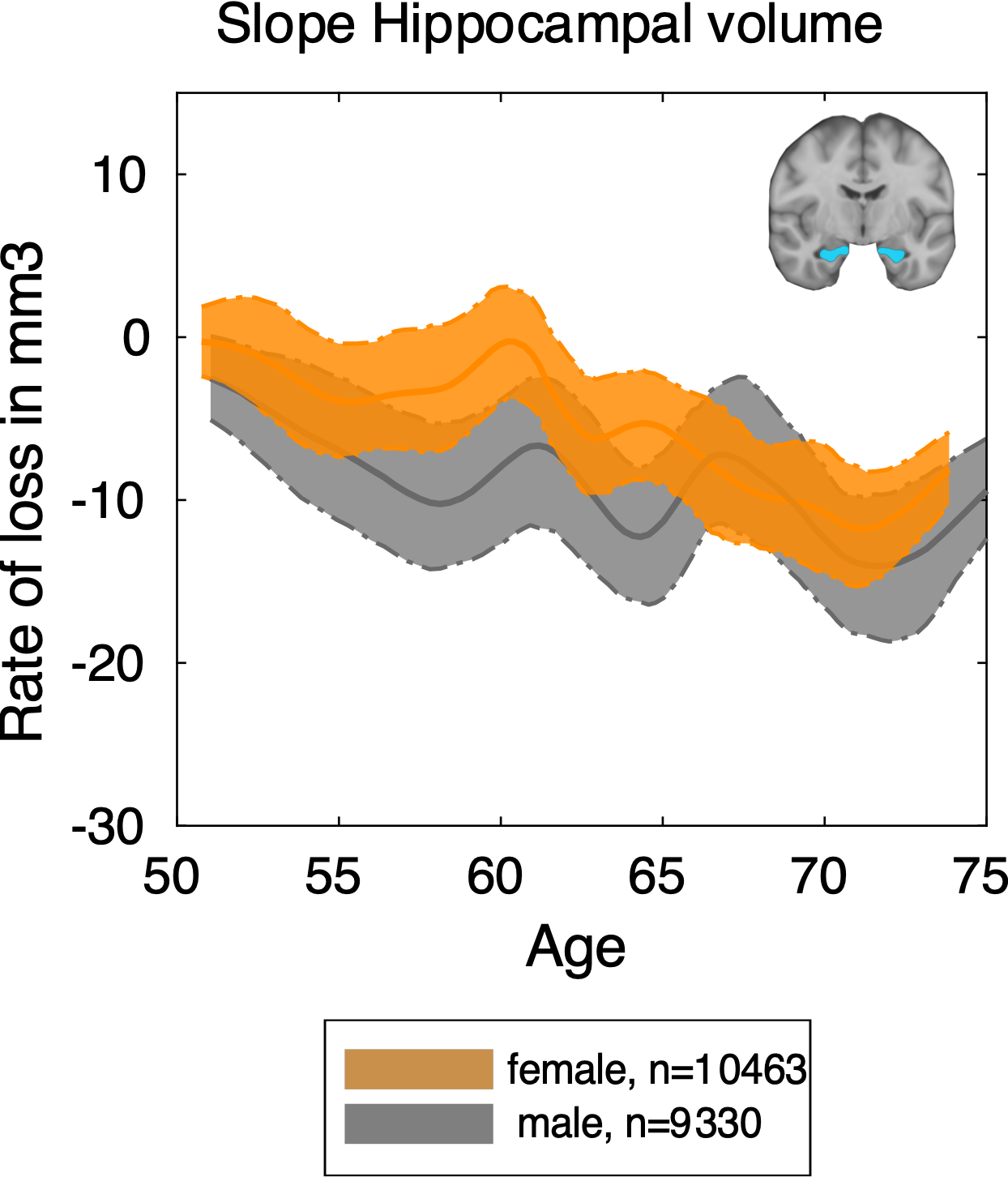


**Suppl. Figure S19**: Mean slope of bilateral hippocampal volume as a function of age, including 95% bootstrapped confidence intervals.


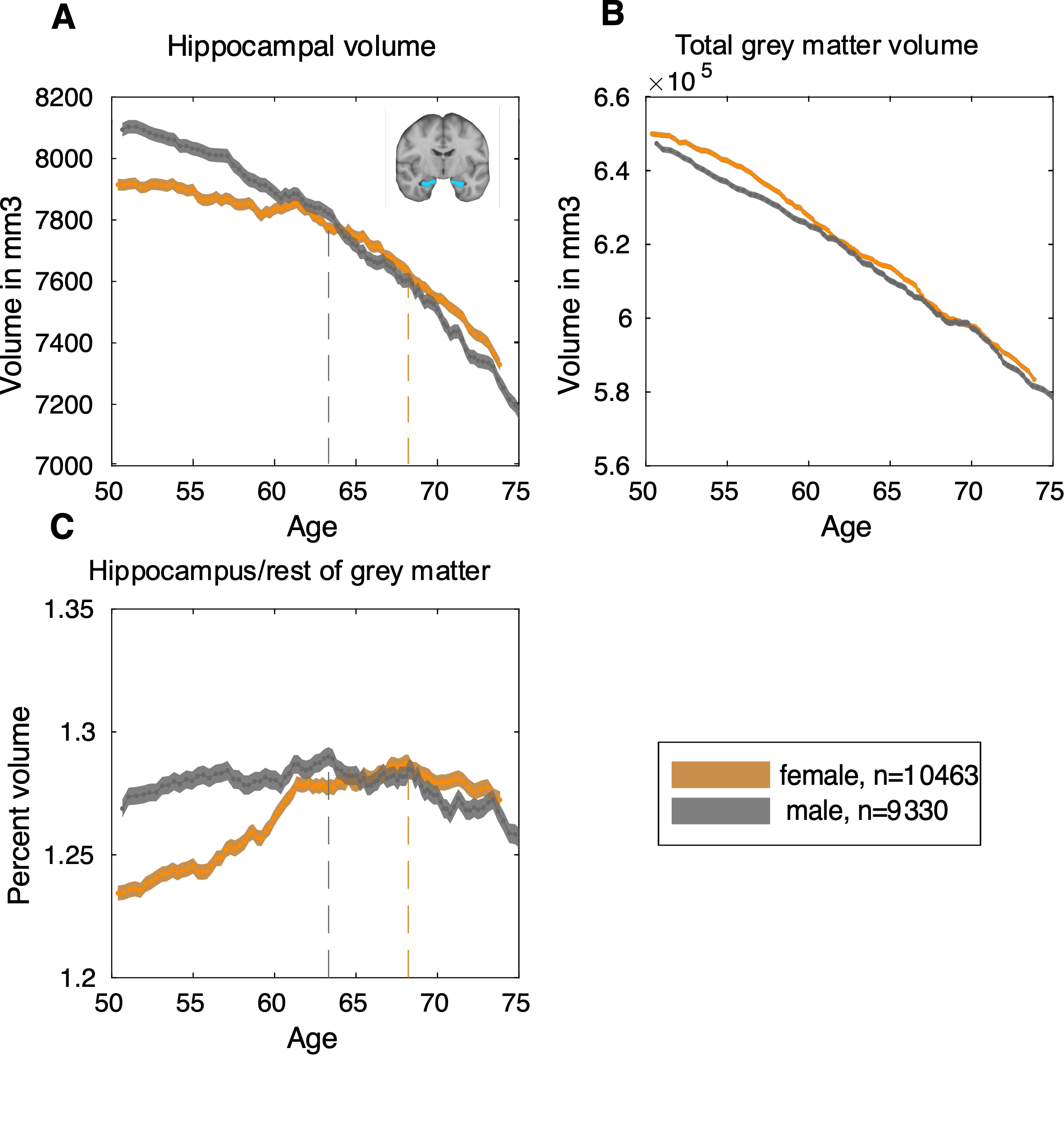


**Suppl. Figure S20:** *Sliding-window curves without smoothing*

Dashed lines indicate points of maximum ratio. **A**. Mean bilateral hippocampal volume including standard errors as a function of age, corrected for head size. **B**. Mean total grey matter volume including standard errors as a function of age, corrected for head size. **C**. Mean hippocampal volume to rest of grey matter ratio including standard errors as a function of age.

**Suppl. Figure S21:** *Sliding-window curves with smoothing kernel of 10*

Dashed lines indicate points of maximum ratio. **A**. Mean bilateral hippocampal volume including standard errors as a function of age, corrected for head size. **B**. Mean total grey matter volume including standard errors as a function of age, corrected for head size. **C**. Mean hippocampal volume to rest of grey matter ratio including standard errors as a function of age.


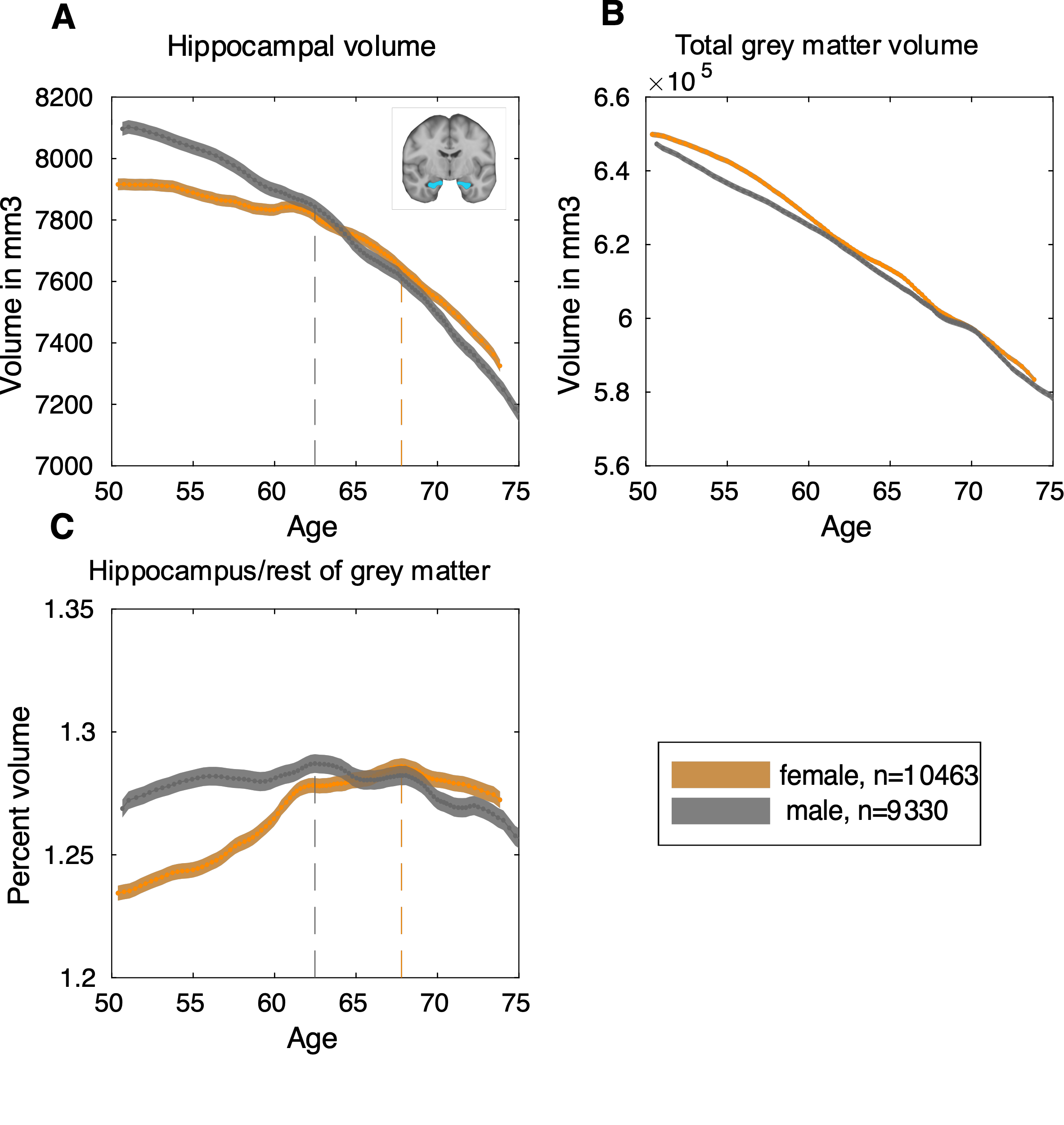


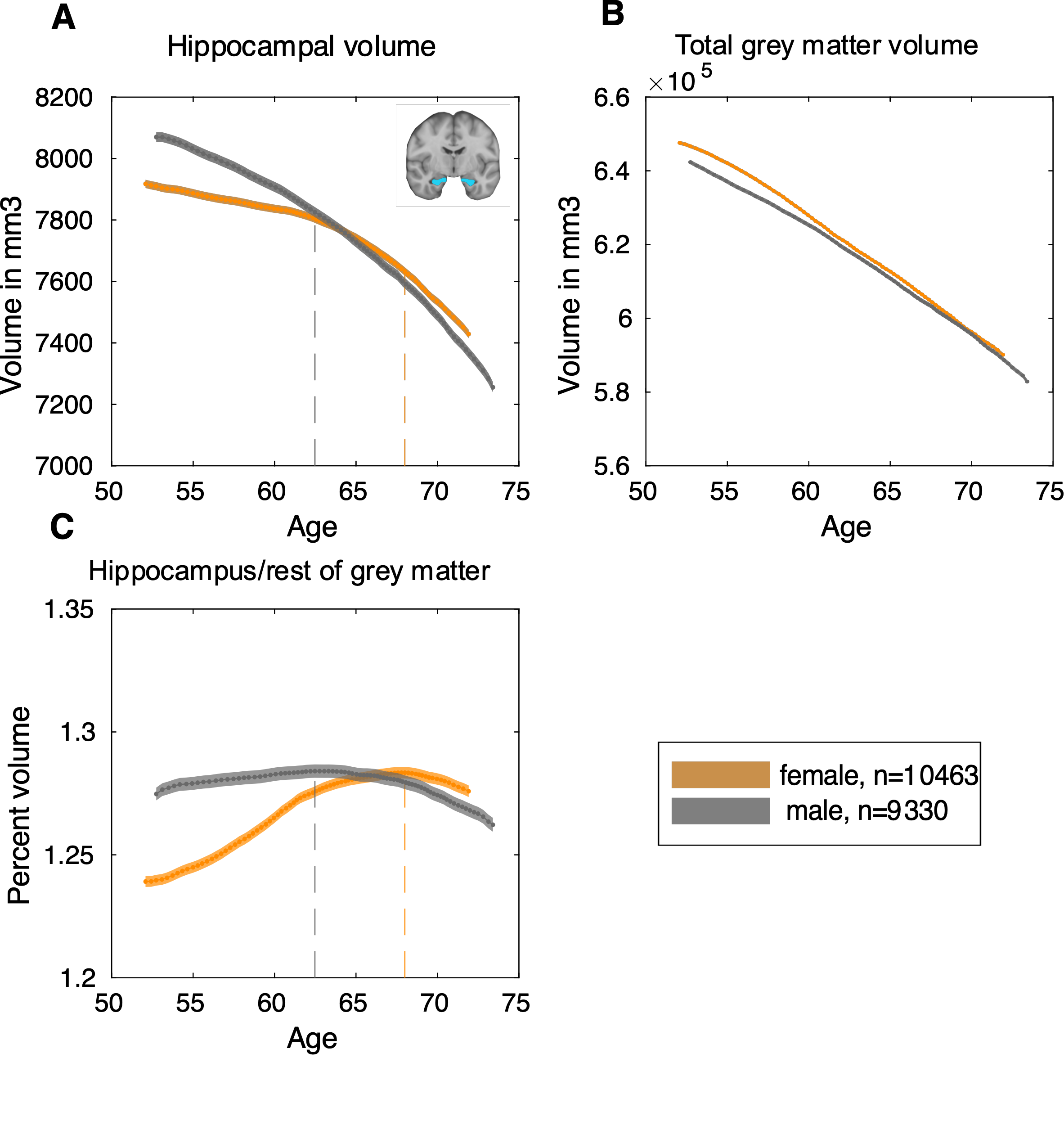


**Suppl. Figure S22:** *Sliding-window curves with 20% quantile width and smoothing kernel of 20*

Dashed lines indicate points of maximum ratio. **A**. Mean bilateral hippocampal volume including standard errors as a function of age, corrected for head size. **B**. Mean total grey matter volume including standard errors as a function of age, corrected for head size. **C**. Mean hippocampal volume to rest of grey matter ratio including standard errors as a function of age.


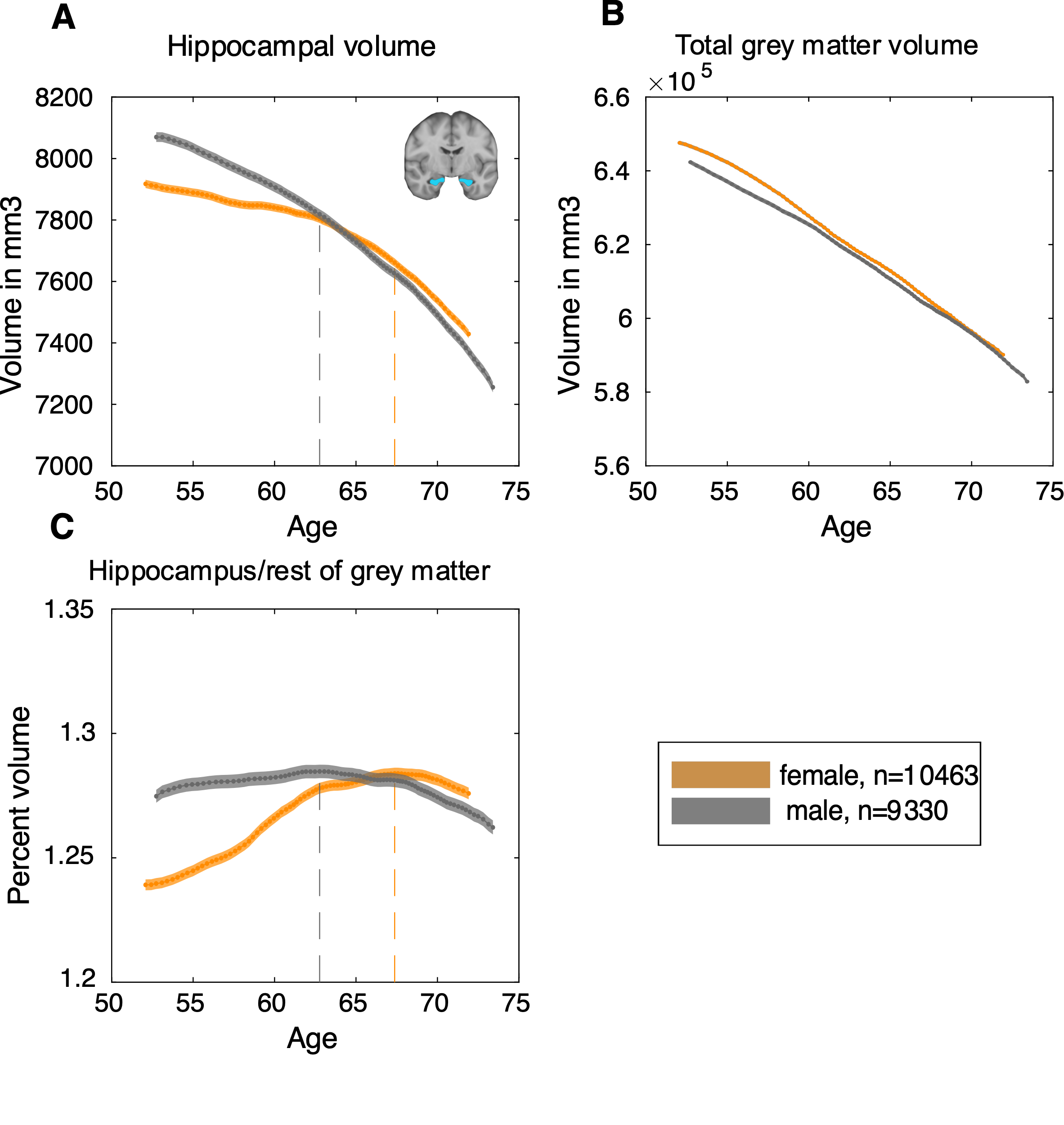


**Suppl. Figure S23:** *Sliding-window curves with quantile width 20% and smoothing kernel of 10*

Dashed lines indicate points of maximum ratio. **A**. Mean bilateral hippocampal volume including standard errors as a function of age, corrected for head size. **B**. Mean total grey matter volume including standard errors as a function of age, corrected for head size. **C**. Mean hippocampal volume to rest of grey matter ratio including standard errors as a function of age.


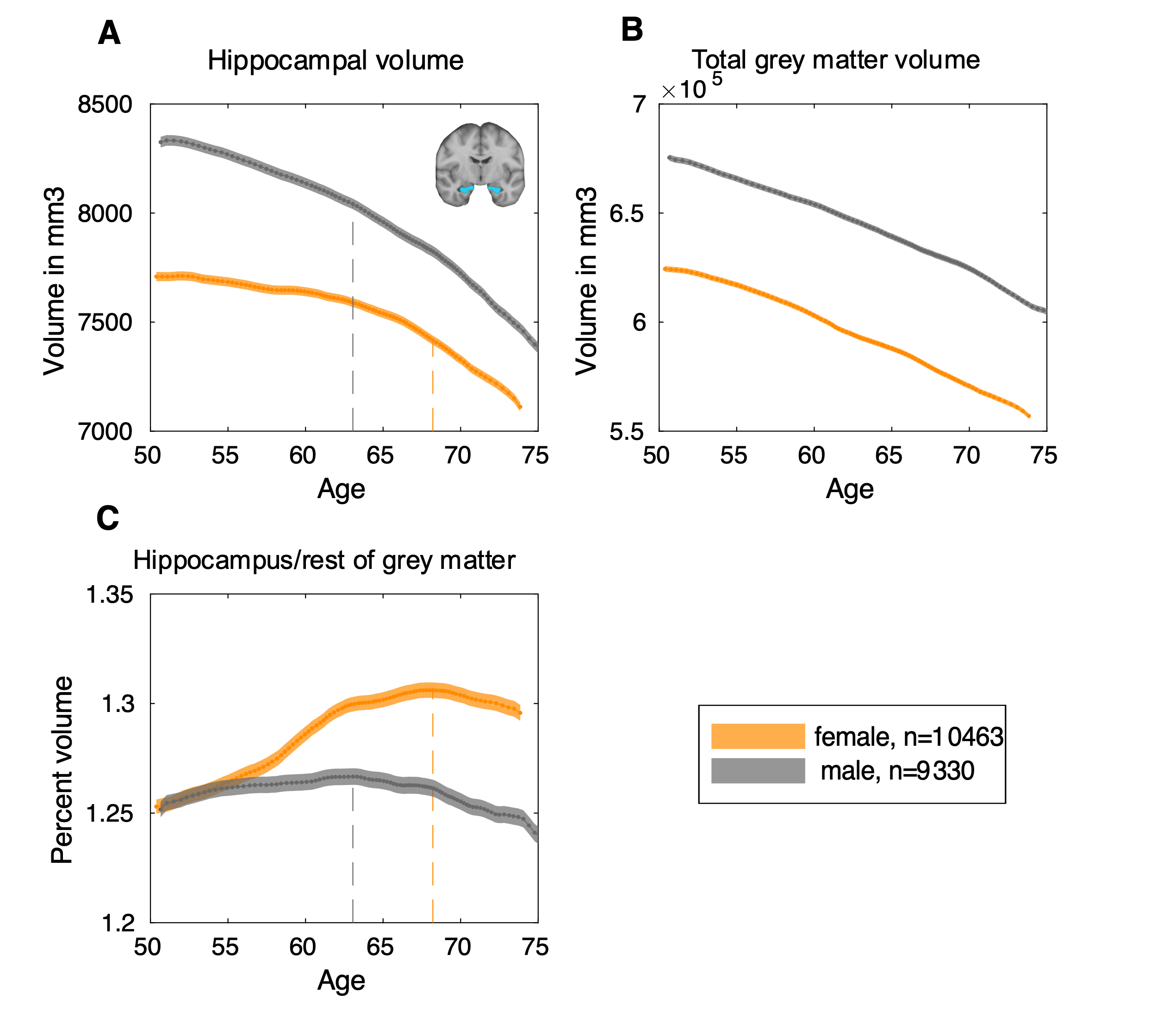


**Suppl. Figure S24:** *Sliding-window curves with quantile width 20% and smoothing kernel of 10, uncorrected for head size*

Dashed lines indicate points of maximum ratio. **A**. Mean bilateral hippocampal volume including standard errors as a function of age, not corrected for head size. **B**. Mean total grey matter volume including standard errors as a function of age, not corrected for head size. **C**. Mean hippocampal volume to rest of grey matter ratio including standard errors as a function of age.
